# IS THERE AN ASSOCIATION BETWEEN GLUCOCORTICOID USE AND FRACTURES? A COMPARATIVE STUDY IN A TRAUMA HOSPITAL

**DOI:** 10.1590/1984-0462/;2019;37;1;00001

**Published:** 2018-07-26

**Authors:** Emanuel Sávio Cavalcanti Sarinho, Verônica Maria Pinho Pessôa Melo, Marcelo Tavares Viana, Marina Maria Pessôa Melo

**Affiliations:** a Universidade Federal de Pernambuco, Recife, PE, Brasil.

**Keywords:** Fractures, Trauma, Glucocorticoids, Children, Adolescents, Fraturas, Trauma, Glicocorticoides, Crianças, Adolescentes

## Abstract

**Objective:**

To assess the association between traumatic fractures and glucocorticoids taken 12 months prior to a trauma, in children and adolescents seen at an emergency room.

**Methods:**

A case-control study was conducted from April to October 2015, at a pediatric emergency hospital with patients aged 3- to 14 years-old, who had suffered physical trauma. Some of the patients had a fracture and some did not. The data analyzed were obtained from medical records, physical examination of the patients, and interview with the patients’ caregivers. Glucocorticoid use in the past 12 months, demographic variables, body mass index, milk intake, trauma intensity, physical activity and smoking in the household were compared between the two patient groups.

**Results:**

A total of 104 patients with physical trauma were studied - 50 had a fracture and 54 did not. Of all the patients, 15.4% had previously used glucocorticoids, and there were no statistically significant differences between the groups. The age range of 10- to 14 years-old, severe trauma and physical activity were more prevalent among patients with a bone fracture.

**Conclusions:**

This study did not find an association between previous glucocorticoid use and the occurrence of fractures in children and adolescents. The age range of 10- to 14 years-old, severe trauma, and physical activity were associated with an increased risk for fractures.

## RESUMO

**Objetivo:**

Avaliar se houve associação entre a ocorrência de fratura após trauma físico e o uso de glicocorticoides nos 12 meses precedentes ao trauma, em crianças e adolescentes atendidos em uma emergência.

**Métodos:**

No período de abril a outubro de 2015 foi conduzido em uma emergência pediátrica um estudo tipo caso controle, em pacientes de 3 a 14 anos incompletos, vitimados por trauma físico, com e sem fratura. Os dados analisados foram obtidos pela consulta dos prontuários, pelo exame físico dos pacientes e por entrevista dos responsáveis, comparando-se uso de glicocorticoides nos últimos 12 meses, características demográficas, índice de massa corpórea, ingesta de leite, intensidade do trauma, prática de exercício físico e tabagismo passivo domiciliar nos dois grupos de pacientes.

**Resultados:**

Estudaram-se 104 pacientes com trauma físico, 50 com fratura e 54 sem fratura. O uso de glicocorticoides ocorreu em 15,4% dos pacientes estudados, sem diferença estatisticamente significante entre os dois grupos. A faixa etária de 10 a 14 anos incompletos, o trauma grave e a prática de exercício físico predominaram entre os pacientes com fratura.

**Conclusões:**

Este estudo não mostrou associação entre o uso prévio de glicocorticoides e a ocorrência de fraturas em crianças e adolescentes. A faixa etária de 10 a 14 anos incompletos, o trauma grave e a prática de exercício físico associaram-se com maior risco para fraturas.

## INTRODUCTION

In the morbidity and mortality profile of children and adolescents, accidents stand out. National data show that 20% of accidents occur with children, 50-60%occur at home and 12.1 to 14.4% cause fractures (second only to falls).^1^ European studies show that 30-50% of young people will suffer from a fracture up until 17 years age.^2^ ^,^ ^3^ ^,^ ^4^ ^,^ ^5^ A series of intrinsic and extrinsic factors, in addition to a trauma, are involved in causing a fracture. It is important to identify whether these fractures signal underlying bone fragility, ie, osteoporosis.

Chronic inflammatory diseases associated with long-term use of glucocorticoids (GCs) are the main causes of secondary osteoporosis.^6^ ^,^ ^7^ ^,^ ^8^ ^,^ ^9^ In childhood and adolescence, these drugs are mainly used for the treatment of asthma, in addition to rheumatologic and autoimmune diseases. The risk of fracture increases considerably after starting continuous corticosteroid therapy and decreases with the same intensity upon discontinuing the treatment.^10^ ^,^ ^11^An excess of these steroids deviates bone remodeling toward reabsorption.^6^ ^,^ ^10^ ^,^ ^12^

Adequate nutrition and the relationship between bone and muscle tissue play a prominent role in bone health. Calcium and vitamin D are fundamental nutritional factors in this regard.^13^ Obesity has a controversial effect, but can act as a mechanical stress on the bone, stimulating mineral and bone mass accumulation.^14^ Genetic, endocrine and mechanical factors simultaneously alter muscle and bone metabolism.^15^ Muscle activity is essential for bone formation and growth.^16^ Among environmental factors, nicotine, the main component of cigarettes, has been associated with the suppression of osteogenesis.^17^

National and regional studies that analyze the association of multiple variables, especially with regard to the use of glucocorticoids and fractures in children and adolescents, can contribute to the establishment of more specific preventive and therapeutic measures. Furthermore, they can reinforce the need for corticoid therapy to be applied more carefully.

This study, therefore, aimed to verify if there is an association between the use of glucocorticoids and fractures in children and adolescents treated in a trauma hospital. In addition, we aimed to compare the demographic and trauma profile, body mass index (BMI), physical exercise, milk intake and household smoking in groups of patients that had and did not have fractures.

## METHOD

In the period from April to October 2015, a case-control study with convenience samples was carried out. Children and adolescents who had suffered a physical trauma, had or did not have fractures, and were attended at the pediatric emergency room of the Hospital da Restauração (Recife, Pernambuco) during that period, were recruited for the investigation. The Research Ethics Committee (*Comitê de Ética em Pesquisa* - CEP) from the hospital approved the research. The search for patients happened twice a week, including on the weekends.

The inclusion criteria were:

1. informed consent, signed by the caregiver and by the patient starting at the age of 12;
2. both genders;
3. an age range from 3 to 14 years old;
4. cases: admission with spontaneous or traumatic fractures, including from a trauma that had a strong impact and multiple traumas (collision, being run over, rolling over);
5. controls: admission with trauma, but without current or past fractures;
6. no primary bone disease, neoplasia, chronic kidney disease, endocrine disease, or malabsorption syndrome.

After selecting the patients, whose age was stratified into three groups (3-7 years old, 7-10 years old and 10-14 years old), a questionnaire designed by the authors was applied using interviews. Demographic (gender, age, ethnicity), social (number of children/adolescents in the household, smoking at home) and accident (location, time, cause) data were recorded. The estimated amount of milk intake (stratified at <200 mL/day and ≥200 mL/ day), the use of glucocorticoids in the last 12 months (dose, duration, frequency and administration method), the presence of an associated disease (specifying it), and regular physical exercise were also checked. Physical exercise was defined as being regular when it was performed under the supervision of an instructor for at least two times a week and for at least three months.

The trauma and the fracture (in the cases) were described in detail. The presence of two or more fractured bones, even if they were on the same side, was considered a multiple fracture. The Landim and Nilsson scale, modified by Clark et al.,^18^ was used to classify the intensity of the trauma.

When patients were immobilized to their bed, measuring body weight was limited. In these cases, the guardian’s knowledge of the last recorded weight was used. However, for the vast majority of patients, body weight and height were measured on a Filizola scale (São Paulo, São Paulo, Brazil), with a coupled anthropometric ruler (model 31, n. 88,401, Indústrias Filizola S/A, São Paulo, São Paulo, Brazil). A metal metric tape measure, attached to the lower and lateral contours of the bed, was used to measure the length of those patients whose height was less than 1 m and who were confined to the bed. BMI was calculated by dividing the weight, in kilograms (kg), by the square of the height, in m, ie. kg/m^2^. The definition of obesity, being overweight, and the risk of being overweight was based on the BMI and Z scores of the World Health Organization (WHO).^19^

The probable embarrassment caused by the Tanner’s pubertal stage evaluation in an emergency room or in an infirmary full of patients and their companions led to the elimination of this examination among the patients studied.

The collected data were gathered in spreadsheets (Excel) and subsequently transferred, tabulated and analyzed by the Statistical Package for the Social Sciences software (SPSS, IBM SPSS Statistics 22, for Windows, 2013). The descriptive analysis was carried out with cross-referenced tables in order to reveal the distribution of percentages and frequencies. For the inferential analysis, the chi-squared test was used to compare the proportion of the dichotomous outcome in the two groups, and the odds ratio (OR), in order to determine the strength of the association with the outcome. All of the ratios had a 95% confidence interval (95%CI). The p<0.05 was considered to be indicative of a significant difference. The multivariate analysis, by logistic regression, was performed to verify the potential effects of the variables on the occurrence of fractures.

## RESULTS

In this study, 104 patients were included, 50 that had experienced a trauma and a fracture (cases) and 54 that had ­experienced a trauma but had no fracture (controls). Of the total, 84 (80.8%) were male and 42 (40.4%) were in the age group of 10 to 14 years old. Table 1 shows the distribution of the main variables studied in the groups that had and did not have a fracture. Table 2 shows the ORs that associate the main variables with the occurrence of a fracture.

**Table 1:** Demographic and clinical characteristics of the patients that did and did not have fractures.

			Fractures		Total	p-value
			Yes	No		
Age Range	3-7 years old	n	9	27	36	<0.001
		%	0.18	0.5	0.34	
	7-10 years old	n	12	14	26	
		%	0.24	0.25	0.25	
	10-14 years old	n	29	13	42	
		%	0.58	0.24	0.40	
Gender	Female	n	7	13	20	
		%	0.14	0.24	0.19	
	Male	n	43	41	84	
		%	0.86	0.75	0.80	
Smoking in the household	Yes	n	21	17	38	0.270
		%	0.42	0.31	0.36	
Milk intake	≥200 mL/day	n	31	35	66	0.768
		%	0.62	0.64	0.63	
Associated disease	Yes	n	12	15	27	0.664
		%	0.24	0.27	0.25	
Previous glucocorticoid use	Yes	n	7	9	16	0.709
		%	0.14	0.16	0.15	
Physical exercise	Yes	n	21	10	31	0.008
		%	0.42	0.18	0.29	
Trauma classification	Light/moderate	n	20	34	54	0.019
		%	0.40	0.62	0.51	
	Severe	n	30	20	50	
		%	0.60	0.37	0.48	
Family history of fractures	Yes	n	31	33	64	0.926
		%	0.62	0.61	0.61	
BMI	Overweight/obese	n	14	14	28	0.446
		%	0.28	0.25	0.26	
Soda intake (mL/day)	≥200 mL/day	n	13	9	22	0.244
		%	0.26	0.16	0.21	

*significant p-value < 0.05; BMI: body mass index.

**Table 2:** Association of risk factors with presence of fractures.

	OR	95%CI
Previous use of glucocorticoids		
Yes	0.840	0.338-2.086
No	1.032	0.876-1.215
Physical Exercise		
Yes	2.268	1.187-0.93
No	0.711	0.544-0.93
Gender		
Female	0.581	0.252-1.339
Male	1.132	0.939-1.365
Milk intake (mL/day)		
<200	1.08	0.650-1.791
≥200	0.956	0.713-1.281
Family history of fractures		
Yes	1.014	0.748-1.374
No	0.977	0.600-1.59
Soda intake (mL/day)		
<200	0.888	0.724-1.087
≥200	1.560	0.731-3.328
Smoking in the household		
Yes	1.334	0.800-2.223
No	0.846	0.628-1.139
Associated disease		
Yes	0.864	0.449-1.662
No	1.052	0.838-1.32
Trauma classification		
Light/moderate	0.635	0.427-0.944
Severe	1.620	1.069-2.453

The systemic use of GCs in the 12 months prior to the trauma was reported by 16 patients (15.4% of the total), of which seven had a fracture (14%) and nine had no fracture (16.7% of the controls). All of the patients had used corticosteroids in intermittent cycles for a maximum duration of seven days (most of them for five days), a cumulative dose of <1 g / year, and mostly to treat asthma attacks. Three of them had used them for different reasons: one for urticaria, one for isolated rhinitis, and one for an upper respiratory tract infection. Only two patients had used continuous inhaled GCs, and one of them was in the fracture group. There was no statistical difference between the groups that did and did not have a fracture in relation to the use of steroids (p = 0.70), nor was there an increase in the risk for fracture in the group that used corticosteroids (OR 0.80 for fractures, 95%CI 0.34-2.09), as demonstrated in Tables 1, 2 and 3.

**Table 3:** Chance of fractures according to previous use of glucocorticoids (GC).

GC use	Fracture			OR	95%CI
	Yes	No	Total		
Yes	07	09	16	0.84	0.338-2.086
No	43	45	88	1.032	0.876-1.210
Total	50	54	104		

OR: odds ratio; 95%CI: 95% confidence interval.

Severe trauma was observed in six of the seven patients that had a fracture and had previously used GC, which could justify the occurrence of the fracture. Falls accounted for 59 of all accidents (56.7%), being run over by a motorcycle for 12, and accidents on a motorcycle for 10, totaling 22 occurrences (21.1%) involving this type vehicle among the patients studied. Accidents on bicycles affected 9 patients (8.6%), and accidents in cars affected 6 (5.8%). Three patients (2.9%) were run over by other vehicles (not motorcycles), animal aggression (being kicked) was reported in 3 individuals, and 2 (1.9%) were hit by a heavy object.

**Figure 1:**
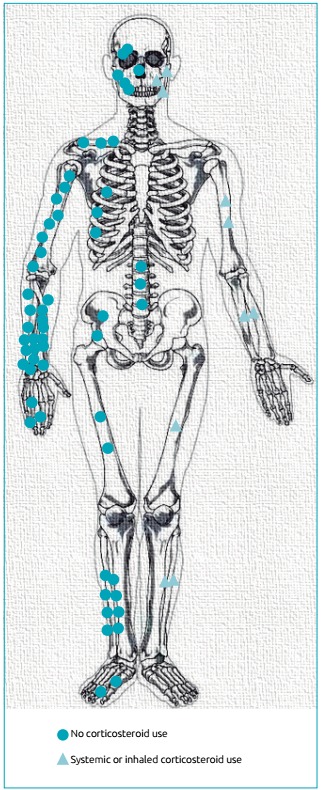
Topography of the fractures among the patients that used glucocorticoids and those that did not. Among the six that used glucocorticoids, three had fractures in two bones. Among the 43 that did not use steroids, 16 had a fracture in more than one bone.

Figure 1 illustrates fracture sites in patients with and without prior GC use. The distal forearm was the site that was most fractured (19 patients in all), corresponding to 38% of the entire sample studied. The humerus was the second most fractured bone. The bones in the face, followed by the humerus, were the most affected among patients who had used GCs. Vertebral fractures occurred in three individuals, but only in the group that did not use GCs.

The BMI assessment showed that 14 of the 50 patients with a fracture (28%) were above the Z +1 score (including being overweight and obese), but only three (6%) were above the Z +2 (obesity) score. Among those that did not have a fracture, 14 also had a BMI above +1 (25.9%), and of these, five (9.3%) were considered obese. The condition of being overweight /obese was recorded in 26.9% of the entire sample studied.

Tables 1 and 2 also demonstrate that only the age group, the intensity of the trauma and the practice of physical exercise showed a statistically significant difference between the patients who did and did not have a fracture (p<0.05). Physical exercise was associated with a double fracture risk (OR for fracture 2.26, 95%CI 1.19-4.33), as described in Table 4. It is worth noting that the fracture occurred during sports practice in 19% of those who practiced regular exercise, and the remaining 81% suffered from a fracture unrelated to the training.

**Table 4:** Chance of fractures according to regular physical activity (RPA).

RPA	Fracture			OR	95%CI
	Yes	No	Total		
Yes	21	10	31	2.268	1.187-4.333
No	29	44	73	0.712	0.544-0.931
Total	50	54	104		

OR: odds ratio; 95%CI: 95% confidence interval.

The multivariate analysis, by logistic regression was performed to verify the potential effects for the occurrence of fractures. It only revealed significance, however, for the age group and trauma severity variables.

## DISCUSSION

Chronic use and intermittent administration of systemic oral GCs have the potential to reduce bone mineral density, increasing the risk for fractures.^12^ However, the occurrence of fractures has been more related to the continuous use of systemic GC for more than three months.^20^ The relative risk of fracture increases with the dose and duration of corticosteroid treatment, but low daily values of prednisolone (2.5 mg) in repeated intermittent cycles may have a cumulative effect that is also harmful.^7^ The GC-induced bone disease mainly affects the trabecular bone, explaining the high number of vertebral and rib fractures.^21^

A systematic review and meta-analysis covering the period from 1966-2013 found a small number of studies relating bone health in children with GCs, thus suggesting a higher prevalence of vertebral morphometric fractures.^22^ Most of these studies evaluated patients with renal and rheumatological diseases that required continuous and prolonged use of the steroids. Our patients, however, only used systemic corticosteroids in intermittent cycles at a frequency of less than five times a year. Nonetheless, a large cohort study in English children found an increased risk of fractures, especially of the humerus, among those who used more than four cycles of oral GCs in the 12 months preceding the fracture.^23^ However, the role of the severity of the underlying disease was not evident, and there was a lack of data on the nutritional status and physical activity of the children evaluated.

The purpose of this study was not to investigate vertebral compressive fractures. Meta-regression of randomized controlled clinical trials in adults found annual odds ratios of vertebral fractures between 2.4 and 8.2% in patients with recent continuous GC use (less than six months).^24^ In the present investigation, bone densitometry was not performed. This examination could add more information about the bone structure of our patients. National research compared the bone mineral density of 16 girls with systemic lupus erythematosus and 32 healthy girls, and did not observe a correlation between the activity of the disease and the use of corticosteroids with the values of densitometry.^25^

The higher frequency of males among the patients studied is also reported in the scientific literature. National and international epidemiological studies are unanimous in relation to the predominance of boys among the injured, and among those who did and did not have fractures. This is probably due to the fact that boys put themselves at risk for accidents more often.^1^ ^,^ ^2^ ^,^ ^3^ ^,^ ^4^ ^,^ ^5^ With regard to the age of the patients that had fractures, more than half were in the age group of 10 to 14 years old. This can be explained by the asynchronism between the pubertal growth spurt and the corresponding accumulation of bone mass, with relative bone weakening, which is also widely reported in the scientific literature.^1^ ^,^ ^2^ ^,^ ^4^

The predominance of falls among pediatric accidents is reported in several studies, ^1^ ^,^ ^3^ ^,^ ^5^ ^,^ ^26^ but it is worth noting that about 25% of the accidents involved motorcycles, a vehicle that is widely used in the region of origin of the patients evaluated, thus requiring specific preventive measures. The reason for the high frequency of severe traumas can be explained by the care profile of the analyzed hospital, which is a state reference in trauma care. However, Clark et al., in a large cohort of children, found that underlying bone fragility was associated with a greater risk for fractures, even in patients who suffered from moderate or severe trauma.^18^

Physical exercise gives greater autonomy to young people. However, despite the fact that it strengthens muscles and bones, it may lead to accidents. Nonetheless, this association, with a double fracture risk, may have been a chance finding, since no significance was observed in the multivariate analysis. On the other hand, a cohort of English children showed that vigorous and daily physical activity increases exposure to injuries and the occurrence of fractures, even when greater volumetric mineral density and bone size are found.^27^

This study had limitations that may compromise its results. The sample should have been 334 patients for an estimated error of 5% and a high sample power. However, our sample, with 104 patients, had a maximum estimated error of 9%. Thus, the population evaluated should have been larger, but the time available and the patients or guardians’ refusal to participate in the research conducted in a trauma emergency room, made it difficult to obtain a greater number of patients. In addition, studies with a case-control design allow for the verification of associations, not necessarily causality. Cohorts with larger samples, followed by a longer study period, are more useful in establishing causal relationships.

Furthermore, the recall bias may have interfered in the results, even though it was minimized by a slow reading of the list of medications. A calibration bias may have occurred at two different moments. First, in the recording of the anthropometric data of the patients that were restricted to their bed and, second, in establishing the actual volume of milk ingested, since it is frequently diluted with water, not to mention there are variations in cup and mug volumes. Additionally, as already mentioned, due to the lack of a space designated for a physical pubertal evaluation, Tanner’s staging was not performed. Finally, the search for vertebral fractures and the evaluation of bone densitometry among the patients studied was not performed due to operational difficulties, though it would add important information to our investigation.

Despite the limitations, this case-control study did not show greater GC use in children and adolescents that had fractures. Probably, a more robust population or cohort study of patients in treatment with repeated cycles of systemic corticosteroids or continuous therapy could better evaluate the risk of fractures.

Adolescents, especially boys, need to receive special attention in order to prevent accidents and fractures, especially with regard to falls and accidents with motorcycles. The practice of physical exercise among children and adolescents requires supervision, in addition to educational instruction to prevent accidents.
